# Adiabatic far-field sub-diffraction imaging

**DOI:** 10.1038/ncomms8942

**Published:** 2015-08-10

**Authors:** Hu Cang, Alessandro Salandrino, Yuan Wang, Xiang Zhang

**Affiliations:** 1 NSF Nanoscale Science and Engineering Center (NSEC), 3112 Etcheverry Hall, University of California, Berkeley, California 94720, USA; 2 Waitt Advanced Biophotonics Center, Salk Institute for Bological Studies, 10010 North Torrey Pines Road, San Diego, California 92037, USA; 3 Materials Sciences Division, Lawrence Berkeley National Laboratory, 1 Cyclotron Road, Berkeley, California 94720, USA; 4 Department of Physics, King Abdulaziz University, Jeddah 21589, Saudi Arabia

## Abstract

The limited resolution of a conventional optical imaging system stems from the fact that the fine feature information of an object is carried by evanescent waves, which exponentially decays in space and thus cannot reach the imaging plane. We introduce here an adiabatic lens, which utilizes a geometrically conformal surface to mediate the interference of slowly decompressed electromagnetic waves at far field to form images. The decompression is satisfying an adiabatic condition, and by bridging the gap between far field and near field, it allows far-field optical systems to project an image of the near-field features directly. Using these designs, we demonstrated the magnification can be up to 20 times and it is possible to achieve sub-50 nm imaging resolution in visible. Our approach provides a means to extend the domain of geometrical optics to a deep sub-wavelength scale.

## Abstract (web summary)

Conventional optical imaging systems are limited in resolution by the decay of the evanescent wave carrying fine feature information. Here, Cang *et al.* propose an adiabatic lens that allows far-field optical systems to project an image of near-field features and achieve sub-50 nm imaging resolution in the visible.

## Graphical abstract

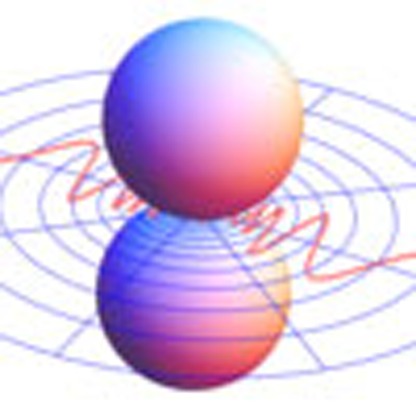

Although a tsunami is devastating near land, it is barely noticeable in the open ocean. This is because the sea floor near the land slows down the tsunami, compressing its wavelength from hundreds of kilometres^1^ to metres and rapidly increasing its amplitude to be destructive. Recently, a similar optical phenomenon has been proposed and demonstrated that surface plasmon waves can, via a similar mechanism, slow down and asymptotically stop light, achieving an ‘optical tsunami'^2^,^3^,^4^,^5^,^6^,^7^,^8^. Remarkably, the variation of the wavelength of the light in the optical tsunami, despite being huge, can be rendered smooth and continuous, or in other words adiabatic. Since the wavelength of light is the fundamental unit that measures the distance between two points in an optical imaging system, the adiabatic variation of the wavelength of light is akin to a ruler with variable units from micrometres to nanometres, the finer end of which one can use to measure sub-diffraction features. Here, we introduce the concept of adiabatic lens (AL) and demonstrate that the optical tsunami can be harnessed to project an image of near-field objects to the far field with sub-diffraction resolution. We present two designs of AL, which, unlike near-field scanning optical microscopes^9^,^10^,^11^, do not need time-consuming raster scan of the sample; and unlike recently proposed meta-materials approaches, do not rely on difficult-to-fabricate negative refractive index^12^ or hyperbolic dispersion^13^,^14^ materials. Rather, the AL utilizes a geometrically conformal surface to mediate the interference of slowly decompressed electromagnetic (EM) waves at far field to form images. AL is a unique device that extends the domain of geometrical optics to deep sub-diffraction length scales. Using these designs, we demonstrated that the magnification can be up to 20 times and it is possible to achieve sub-50 nm imaging resolution in visible.

The difficulty to achieve sub-diffraction imaging reflects the giant mismatch between the length scale of optical wavelength (hundreds of nanometres), and nanometre scale objects of interest. According to Abbe's diffraction theory of imaging, projecting an image of an object requires interference of various components of electromagnetic waves scattered or emitted from the object. Therefore, in an optical system, the wavelength of light sets the fundamental limit in resolving the distance between two points. As such, conventional lenses or mirrors, whose working wavelength is limited by the refractive index of natural materials, cannot image deep sub-wavelength features directly. On the other hand, the optical near field of an object contains high-resolution information, but is confined within nanometres range from the object and is inaccessible to far-field optical devices. While nanometre scale probes have been used in near-field scanning optical microscopes^9^,^10^,^11^ to couple the near-field EM waves to far field via a scattering process, the phase of the near-field EM wave is not preserved, but can only be extracted interferometrically; hence, the scattered light cannot project an image directly and a time-consuming raster scan of the sample is required as a mechanism of image reconstruction. We propose here to utilize adiabatically decompressed plasmon polaritons to bridge the gap between far field and near field.

Recent progress in plasmonics has enabled unprecedented control over light propagation. Adiabatic concentration of plasmon polaritions has been proposed and demonstrated in tapered plasmonic wave guides, which utilize the proximity effects of a second surface near the plasmon-guiding interface to stop light and asymptotically compress the wavelength to near zero^2^,^3^,^6^,^8^,^15^. However, none of these schemes lends itself to imaging applications. We propose here to achieve sub-diffraction imaging by utilizing a reversed, decompression process, whereby the wavelength of the plasmon polariton increases from nanoscale to the vacuum wavelength of light. The decompression is slow and satisfying an adiabatic condition; therefore it is equivalent to a slow variation in the effective refractive index from extremely high to ordinary values. By bridging the gap between far field and near field, this adiabatic decompression allows far-field optical systems to project an image of the near-field features directly. Notice that the required slow variation of the effective index implies that the plasmonic structures considered here must be large in terms of wavelengths, as opposed to other systems^5^, which are sub-wavelength and described electrostatically.

## Results

### A double-sphere design of adiabatic lens

**Figure 1:**
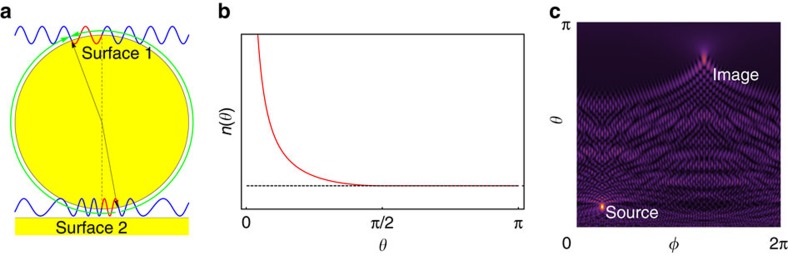
Adiabatic decompression of plasmon polariton for sub-diffraction imaging. (**a**). A cross-section of a double-surface system. The nanometre-sized gap between two metallic surfaces compresses the local wavelength down to the nanometre scale. The wavelength of the EM waves originated from a point source in the gap near the South Pole of the sphere is adiabatically stretched as they are propagating towards the North Pole. Notice how 1*λ* near the North Pole corresponds to larger physical distance compared with 1*λ* near the South Pole. Such dilation of the wavelength gives rise to a pre-magnification that can be exploited for sub-diffraction imaging. (**b**) Plasmonic decompression: the surface waves propagating on the sphere undergo wavelength dilation, going from a high-index gap plasmon to a single-interface plasmon polariton regime. The functional dependence of the plasmon effective index depends on the choice of the second surface. In this specific example we consider a 1/sin dependence. (**c**) Computed image formation (for a 1/sin system) of a point source located at *θ*=*π*/10, *φ*=*π*/4. The image is formed on the upper hemisphere at *θ*≈(7/8)*π*, *φ=3π*/4. As a consequence of the plasmonic decompression, the image is displaced from the North Pole by a larger angular distance compared to the source offset from the South pole.

**Figure 2:**
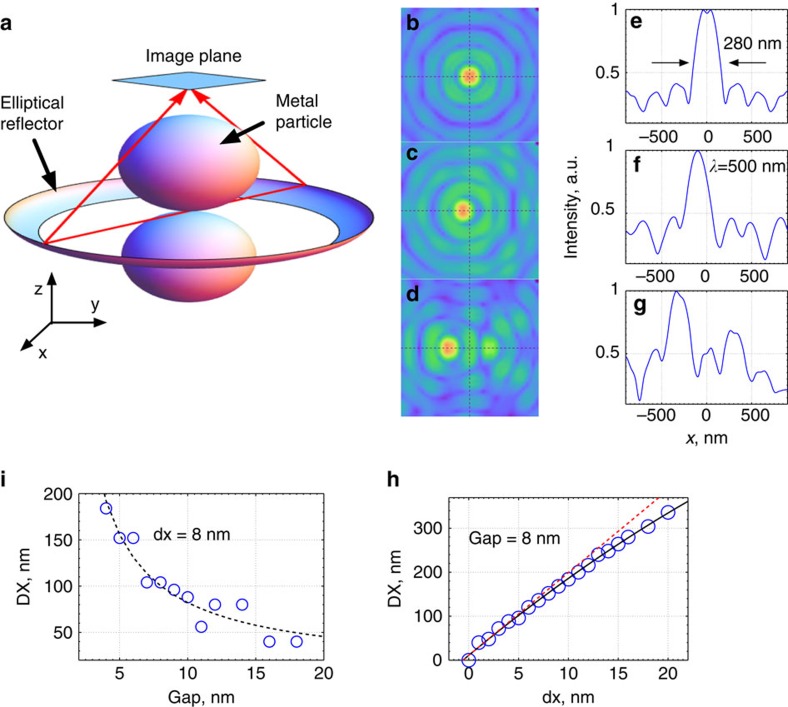
A far-field nanoscope. (**a**) An elliptical reflector (*x*-, *y*- and *z*-radii of 20, 20 and 8 μm, respectively) is placed surrounding a pair of ellipsoidal metal dimers (*x*-, *y*- and *z*-radii of 1.25, 1.25 and 0.25 μm, respectively), forming an imaging system. The ellipsoid dimer was chosen for fewer requirements on computation resources than spherical dimer. The frequency of the light corresponds to 500 nm in vacuum. The centre of the dimer is coincident with one focus of the elliptical reflector. An image plate is placed at the other focus of the elliptical reflector. A point source is placed at the centre of the gap. Its image is shown in **b**. As we move the source horizontally by 8 and 24 nm from the centre, the corresponding images show resolvable movement (**c**,**d**). The corresponding cross-section plots are shown in **e** through **g**. The magnification is plotted in **h** as DX versus dx curve. This calibration is fairly linear within ∼12 nm (red dashed curve). A second order curve (black curve) shows a better fit. (**i**) The resolution depends on the size of the gap. We fix a point source at 8 nm, sweep the gap, and plot the displacement of the image in **i**. A 1/*x* curve describes the DX versus gap relation well, black dash line in **i**.

Figure 1a presents a realization of the adiabatic lens in the form of a double-surface structure. A metallic sphere is placed in nanometre proximity of a second smooth metallic surface. From an electromagnetic point of view, the system morphs from a tapered metal–insulator–metal waveguide^15^,^16^,^17^ in the gap region, to a single plasmonic interface in the upper hemisphere. As the surface plasmon waves originated from an object in the gap region propagate along the curved interface, from the gap to the upper hemisphere, they experience adiabatic decompression; the effective wavelength continuously increases from single nanometres in the gap region to the vacuum wavelength of hundreds of nanometres. The structure shown in Fig. 1a supports both quasi-bound and radiation modes. A source placed in proximity of the metallic surface is capable of exciting two kinds of modes, naturally suggesting two different imaging approaches. One utilizes the interference of the plasmon polariton on the surface of the metallic sphere (Fig. 1); the other approach uses an elliptical reflector to interfere the decompressed EM wave to form an image on a far-field imaging plate (Fig. 2).

We emphasize that the super-resolution is achieved not through a sub-diffraction point-spread function^16^,^17^,^18^, which may require a perfect drain to be realized^19^, but through a giant magnification derived from the decompression of EM waves. In an imaging system shown in Fig. 1a, the wavelength is the fundamental unit that measures the distance between two points. The proposed double-surface system does not have a fixed global wavelength but a spatial grid of variable wavelengths, depending on the distance between the two interfaces. The optical distance between two object points is preserved in image space, when measured in terms of the local wavelength. Because of the adiabatic decompression, that preserves the phase information, the physical distance between the two image points will be magnified as a consequence of the local wavelength dilation from the object to the image space (Fig. 1a and Supplementary Fig. 1).

### Mechanism of super-resolution

To elucidate the mechanism of the super-resolution imaging process, we developed an analytical model for a double-interface plasmonic system. It has been demonstrated that on a single sphere in isolation, surface waves generated by a point source can be refocused at the antipodal point forming an image of the source^16^,^17^,^18^. This is a consequence of the constant positive intrinsic curvature of this geometry. However, the resolution in this case is limited by the wavelength of the surface wave, unless a perfect drain is placed at the imaging point^19^. If the metallic sphere is in contact at one point with a second metallic interface, in the absence of losses and nonlocal effects, the effective index would diverge at the point of contact^5^. The effective index is continuous and monotonic function of the distance between the two interfaces, and decreases as the surface mode is transformed from a gap plasmon to a single-interface surface plasmon polariton (Fig. 1b). Such behaviour can be described in terms of the following equation (Supplementary Note 1) for a disturbance propagating on a spherical surface of radius *R*>>*λ* and under the adiabatic condition ∂*k*_*s*_/∂_*s*_*<<k*_*s*_/λ:


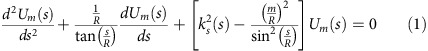


An azimuthal dependence of the form *e*^*imφ*^ is assumed. The surface coordinate *s* is simply related to the polar angle *θ* through *s*=*Rθ*. The function *k*_*s*_(*s*) represents a coordinate-dependent propagation vector tangent to the surface, which accounts for the variation of the local index as a consequence of the geometric potential induced by the interaction between the two spheres. A general solution to equation (1) is not readily available, nevertheless simple closed form solutions can be found for a few specific *k*_*s*_(*s*) profiles to illustrate the concept of the adiabatic lens. In the case of a single sphere, the wave vector *k*_p_ approaches the value of a plasmon at a single interface, and equation (1) reduces to the well-known-associated Legendre's equation. In this case the solution is given in terms of associated Legendre's functions of the first kind. More interesting is a profile of the form:


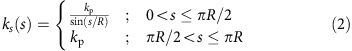


Notice that the specific profile (equation (2)) retains the properties of the local refractive index increasing monotonically from the common plasmon value *k*_p_ to a positive singularity at the contact at *s*=0. The solution is then amenable to the following closed form:


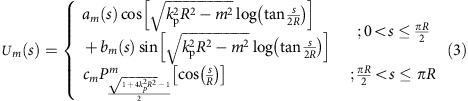


Corrections to the field amplitude stemming from the wave decompression can be obtained by considering the radial dependence of the solutions and imposing power-flow conservation. Equation (1) can be cast in the canonical Sturm–Liouville form; from the knowledge of the set (3) it is possible to obtain the Green's function of the system (Supplementary Note 3), which yields the field distribution in the case of point source excitation shown in Fig. 1c. A point source placed in the lower hemisphere at some angular distance *θ*_0_ from the contact point at the South Pole (*s=0*), launches a surface wave that is refocused in the upper hemisphere at an angular position *θ*_1_>*θ*_0_ from the North Pole (*s=π*/2). This is a direct consequence of the wavelength dilation and effectively results in an angle-dependent magnification effect along *θ.*

This analytical solution elucidates an important aspect of the proposed imaging system: even though the smallest length scale of the system is far below the vacuum wavelength of the light, at every point in space, the system works above the local wavelength, meaning that a Hamiltonian optics formulation can be used to describe the system in terms conventional rays orthogonal to the phase fronts, in contradistinction with the sub-wavelength ‘Poynting vector rays' characteristic of hyperbolic metamaterials^20^. In particular the index profile associated with equation (2) yields geodesics trajectories in the following analytical form:


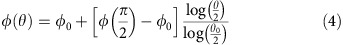


The parameter *φ*_0_ is the azimuthal coordinate at the source point located at elevation *θ*_0_. The subset of the geodesics (4) contributing to the formation of an image is illustrated in the Supplementary Fig. 2, along with the ray envelope (shown in yellow) that encloses them.

Although the profile (2) captures qualitatively well the physics involved in the image formation in the type of systems described here, we would like to stress that the principles outlined are not related to a specific geometry, but are in fact applicable to a more general class of systems supporting guided modes and meeting an adiabatic condition for the variation of the local effective index. We performed full-wave numerical simulations to prove the proposed super-resolution imaging mechanism in the case of two spheres, which are not in contact in the Supplementary Fig. 3.

### Integration of adiabatic lens with a conventional optical system

The approach described so far relies on the guided surface modes excited by a source located in the gap between two metallic interfaces. A complementary mode of operation exploits the scattered angular spectrum instead, and can be integrated with conventional imaging systems. Exploiting an elliptical reflector, here we show in fact that a double-sphere plasmonic system can enhance the resolution of a conventional optics system directly (Fig. 2). One focus of the ellipsoid reflector coincides with the centre of the gap between the two spheres. An imaging plane is placed at the other focus of the elliptical reflector. A point dipole is placed within the gap and emits light in all directions. Our simulation shows that, along each direction, the light experiences adiabatic decompression. The elliptical reflector collects the lights, and interfere them at the image plate. If the light source is at the centre of the gap, all of the light paths will accumulate equal phase at the centre of the image plane. The constructive interference gives rise to a point image (Fig. 2b). If we move the light source away from the centre, light rays along different directions accumulate different phases. The point image will move away from the centre point accordingly (Fig. 2c,d). A displacement of a point source in the gap as small as a few nanometres is clearly resolvable at the image plate, which demonstrates the super-resolution of the system. The resolution depends on the shortest local wavelength that the system can achieve in the gap; therefore decreasing the gap size increases the resolution (Fig. 2i–k).

**Figure 3:**
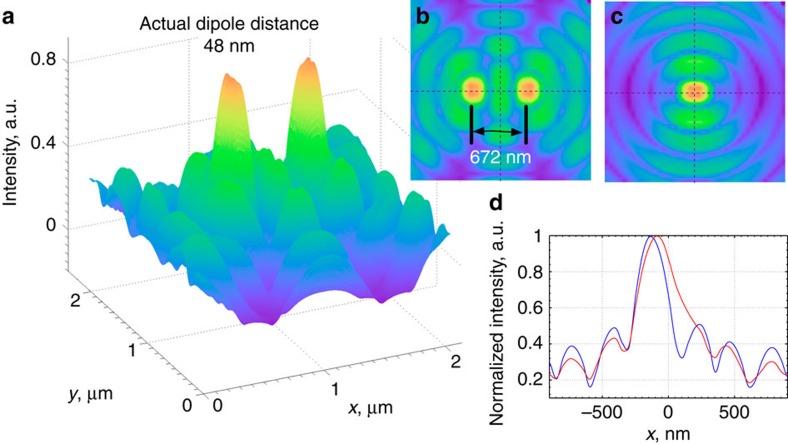
Imaging of two dipole sources. (**a**,**b**) The corresponding far-field images of two point dipoles that are located at 24 and −24 nm, respectively, from the origin within the gap are clearly resolvable with a separation of 672 nm, (**c**) when increase the gap size to 24 nm, the resolution decreases and the two image points cannot be resolved. (**d**) The loss of the metal is evaluated. The blue curve represents a cross-section of an image of a dipole located 8 nm from the centre with a lossless metal. The red curve corresponds to the dielectric constants of −2+*i*. The cross-section widens and resolution reduces, and the centre shifts slightly towards the origin.

In Fig. 3a,b we show that two coherent point sources located 48 nm apart can clearly be resolved at the image plate. As the gap increases to 24 nm, the two points are no longer resolvable (Fig. 3c). The resolution limit of this type of system depends on the extent to which one can slow down the light, and it is ultimately determined by the non-local effect (Supplementary Fig. 4 and Supplementary Note 5) and loss (Supplementary Fig. 5 and 6, Supplementary Note 4) of the material. As shown in Fig. 3e, the loss reduces the magnification as well as increases the width of the image peak. Therefore, we estimate that the practical limit of the resolution that the adiabatic lens can achieve will be limited to the skin depth of the material that is used, which is about tens of nanometres in visible range. A possible method to further circumvent this limit could be by using active materials that uses gain media to compensate the loss^20^,^21^,^22^,^23^,^24^.

## Discussion

Transformation optics provides another angle to understand the origin of the super-resolution in this adiabatic lens^25^,^26^. The second surface provides space curvature that leads to an effective magnification for the field distribution in the gap. Notice however that our system is essentially different from other systems based on transformation optics such as the kissing spheres and cylinders^3^,^4^,^5^, which are not suitable for imaging applications, being themselves of sub-wavelength dimensions. Our geometry on the other hand is much larger than the wavelength and operates based on propagating surface plasmons, rather than resonant modes of the whole structure described in references^3^,^4^,^5^.

Wang *et al.*^27^ have presented an elegant design of a far-field super lens made of gold-coated dielectric microspheres achieving a 50 nm lateral resolution. Its mechanism of super-resolution is fundamentally different from the proposed AL here. Unlike Wang's design, where the metal–dielectric interface contributes to the super-resolution, AL utilizes slowly increased gap between two metal surfaces to adiabatically decompress EM waves and bridge near and far field. One may have noticed that the mechanism of the image formation in this design follows exactly geometrical optics principles. Indeed, in the adiabatic process, the concept of geometrical optics, such as optical path still applies. Geometrical optics has played a pivotal role in science. From telescopes to microscopes, optical instruments designed by geometrical optics have contributed enormously to our knowledge of nature at both the micro- and macro-length scales^28^. Here, our design demonstrates that by utilizing the adiabatic decompression and abandoning global wavelength in favour of a spatial grid of variable wavelengths would provide a means to extend the domain of geometrical optics to a deep sub-wavelength scale. This can provide a novel paradigm in optical design, leading to structures that provide sub-diffraction performance and integrability with conventional optical systems. Moreover, the concept of adiabatic decompression is quite general in nature; and one of the most famous examples perhaps is a tsunami^29^. We envision that the principle found here could be applied beyond the electromagnetic waves, to acoustic waves as well.

## Methods

### Numerical simulations

We use a hybrid method-of-moment with physical optics software (FEKO 6.0) for numerical simulation (Figs 2, 3). Method-of-moment simulates the near-field adiabatic decompression process within the gap between the two ellipsoid particles. Physical optics simulates the interference of propagating wave, which is reflected from the ellipsoid reflector and form images at the imaging plate. To further help visualize this super-resolution imaging process; a supplementary animation (Supplementary Movie) is created by a finite-difference-time-domain simulation with CST Studio showing how the surface wave interferes to form a super-resolution image (Supplementary Note 2).

## Additional information

**How to cite this article:** Cang, H. *et al.* Adiabatic far-field sub-diffraction imaging. *Nat. Commun.* 6:7942 doi: 10.1038/ncomms8942 (2015).

## Supplementary Material

Supplementary Information Supplementary Figures 1-6, Supplementary Notes 1-5 and Supplementary References

Supplementary Movie 1 This video illustrates how surface waves interfere and form a super-resolution image of a point source. The structure consists of a lossless metal sphere (dielectric constant of ε=-2+0.01i, and permittivity μ=1) with diameter of 2μm placed 5nm above an infinite large lossless metal plate. A point dipole source is placed in the gap, 16nm away in X direction away from the center of the gap. The polarization of the dipole is in Z direction. A Finite-Time-Domain-Difference (FDTD) method is used for the simulation. The highest frequency used in the simulation corresponds to light with wavelength of 500nm in vacuum. The video shows a XZ cross-section plot of the intensity of all polarizations components of the E field. One can see that the surface waves propagate from the bottom of the sphere to the top. A white line marks the North Pole. The most constructive interference appears near the North Pole, forms an image of the point source. One may also notice that the adiabatic decompression process from the increasing wavelength of the surface waves. Even though the point source is only 16nm away from the South Pole of the sphere, which is well below the resolution limit. The displacement of this point source is clearly resolvable at the North Pole, illustrating the super-resolution power.
