# Supplementary material for: Adiabatic far-field sub-diffraction imaging
Source: Nat Commun. 2015 Aug 10;6:7942. doi: 10.1038/ncomms8942 (PMC4839783; doi:10.1038/ncomms8942)
Supplement: Supplementary Information — Supplementary Figures 1-6, Supplementary Notes 1-5 and Supplementary References [file ncomms8942-s1.pdf]

Supplementary Online Material
Supplementary Fig. 1:

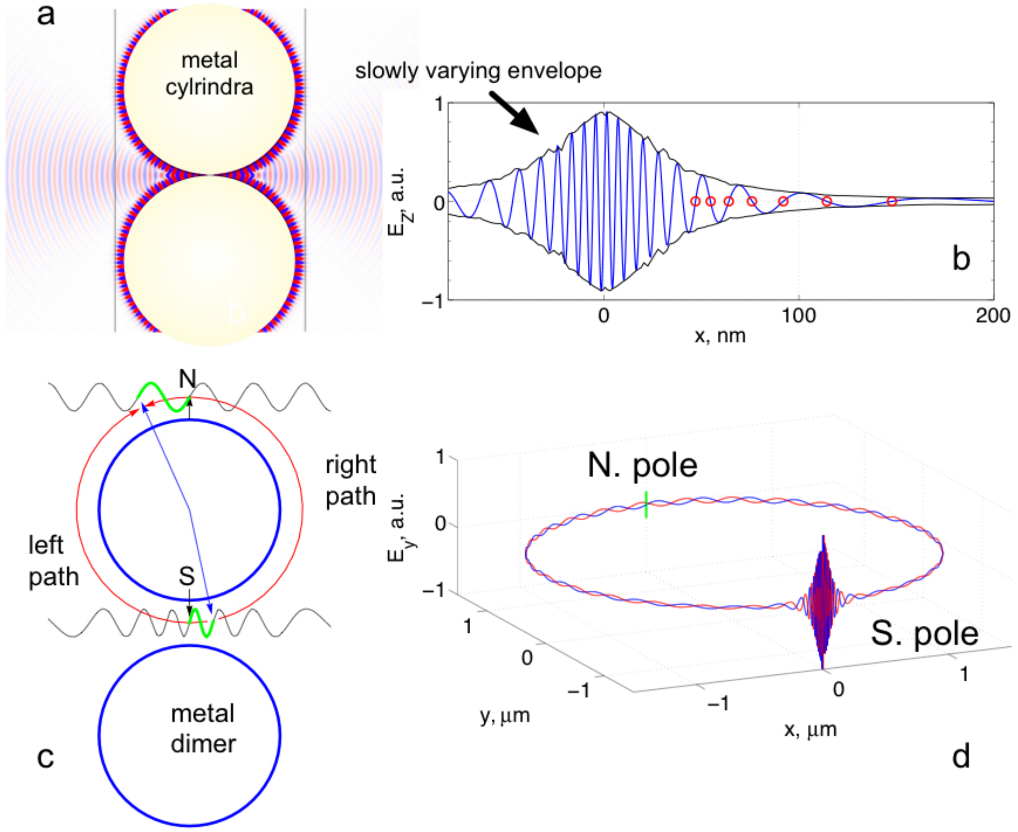

Adiabatic Decompression in 2D: (a). Two metal cylinders of 10 μm in radius, are juxtaposed together, forming a gap of 2 nm in between. A dipole oscillating at a frequency equivalent to a wavelength of 500 nm in vacuum is placed at the center of the gap. The metal is lossless, (dielectric constant of -2+0.001*i*). Maxwell's equations are solved with a finite-element method (FEM); the electric field is plotted in the figure. **(b)** A zoomed-in view of the E-field along *x*-axis is plotted as the blue curve, with the envelop $e(x)$ as the black curve. The chirping is evident, which suggests a possibility of super-resolution **(c-d)**. A dipole located in the gap excites surface waves on the cylinders. The surface waves form interference pattern at the North Pole of the top cylinder, (red curve in **(d)**). If we move the dipole by one *local wavelength*, 5nm, at the South Pole, the pattern will change one *local wavelength*, 250nm, accordingly at the North Pole, shown as the blue curve in (d). Hence, nanometer level displacement of the point source is magnified and become resolvable.

Supplementary Fig. 2:
A Geometrical Optical Interpretation of the Double-sphere System

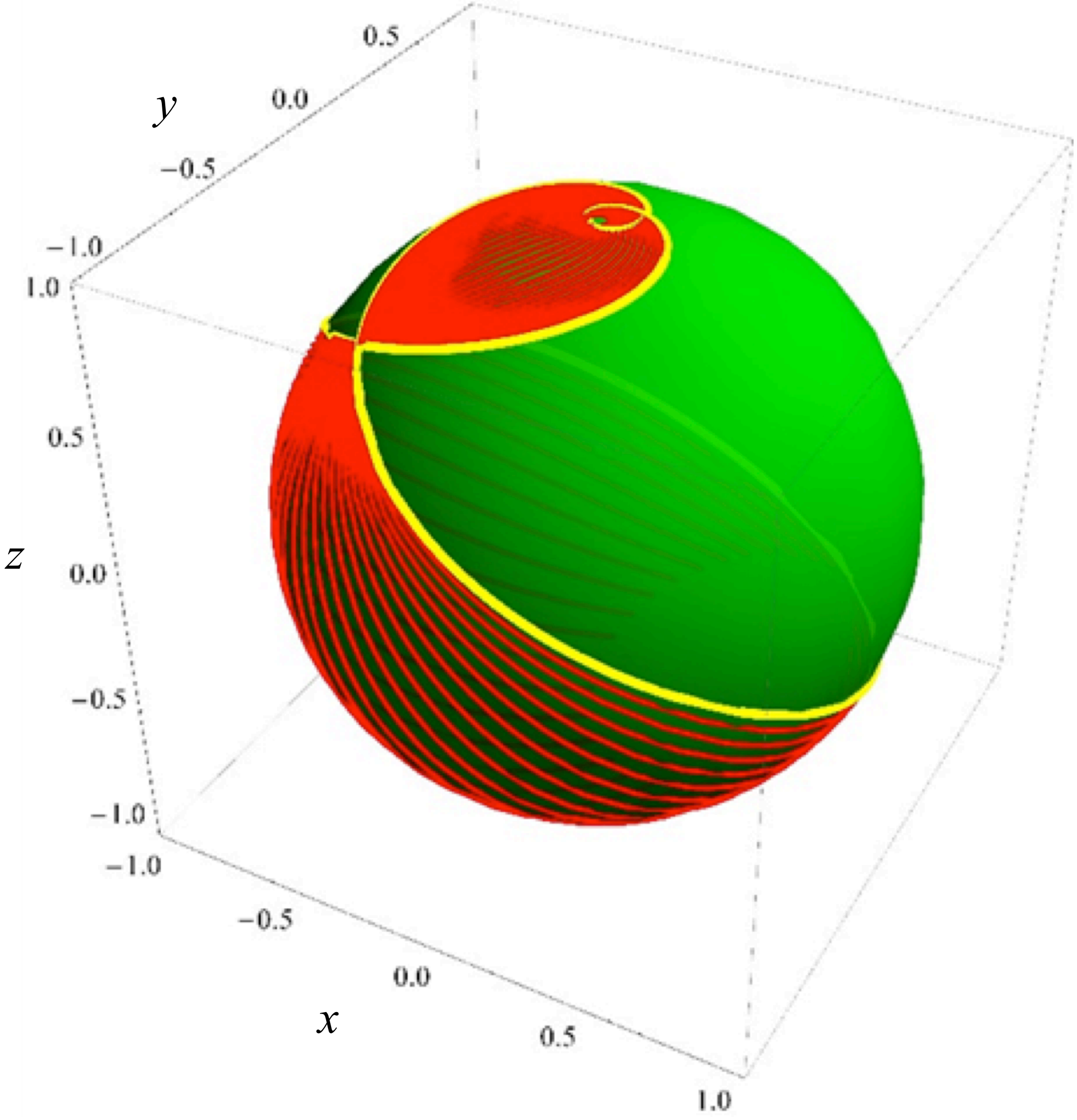

Supplementary Fig. 3:

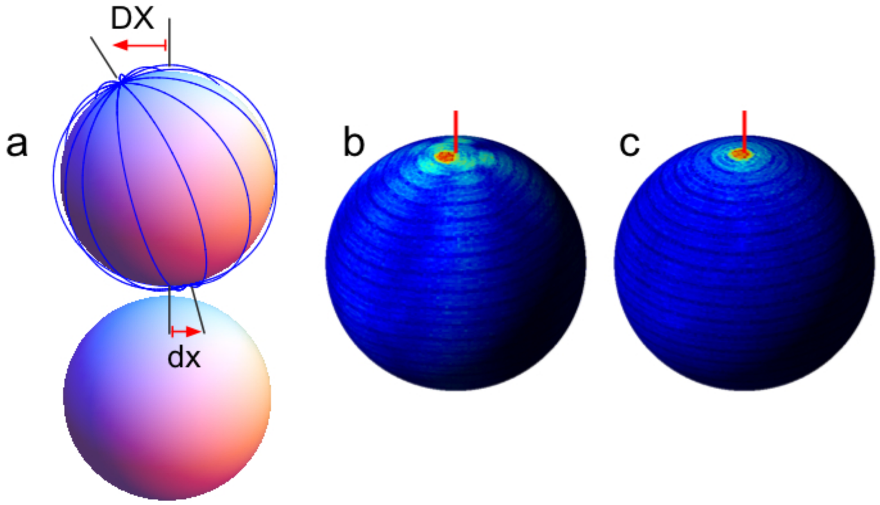

Super-resolution imaging in a double-sphere system: (a) A 3D example consists of two metal spheres (dielectric constant of -2+0.001i and radius of 1.25 μm). The surface wave excited by a point dipole located in the gap between two metal spheres generates an image near the North Pole. Maxwell's equation is solved with a method-of-moment (MoM) calculation, and the intensity of the electric field is plotted on the surface of the top sphere (b-c). In panel (b), the point source is located 8 nm away from the South Pole. Its image formed near the North Pole shifts from the North Pole (red line), and the shift is clearly resolvable. The super-resolution depends on the size of the gap. The gap size is 5 nm in panel (b). We then increase the gap size to 16 nm. As shown in panel (c), the super-resolution disappears.

Supplementary Fig. 4:
Nonlocal response in a 2D double sphere system.

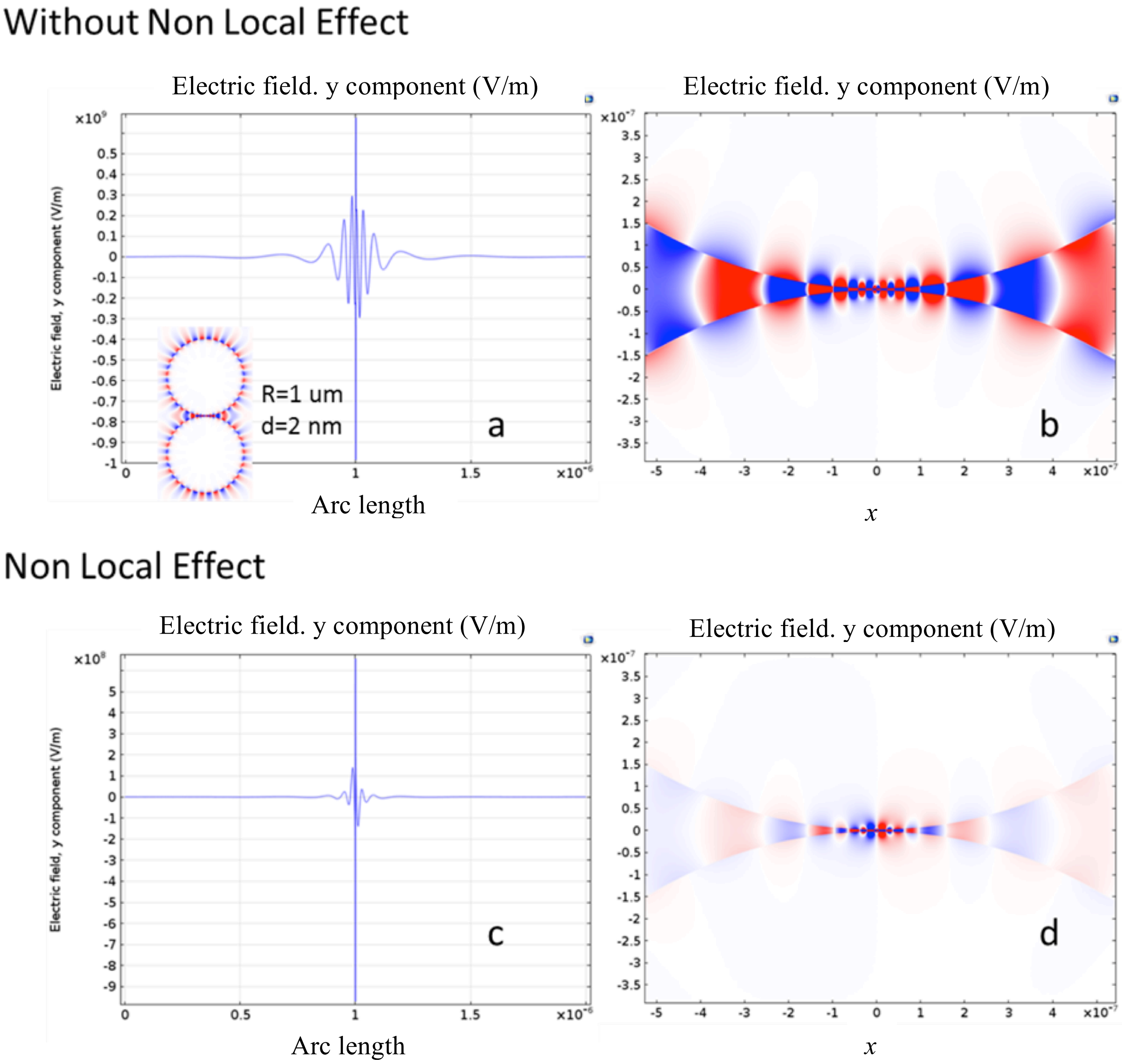

Supplementary Fig. 5:

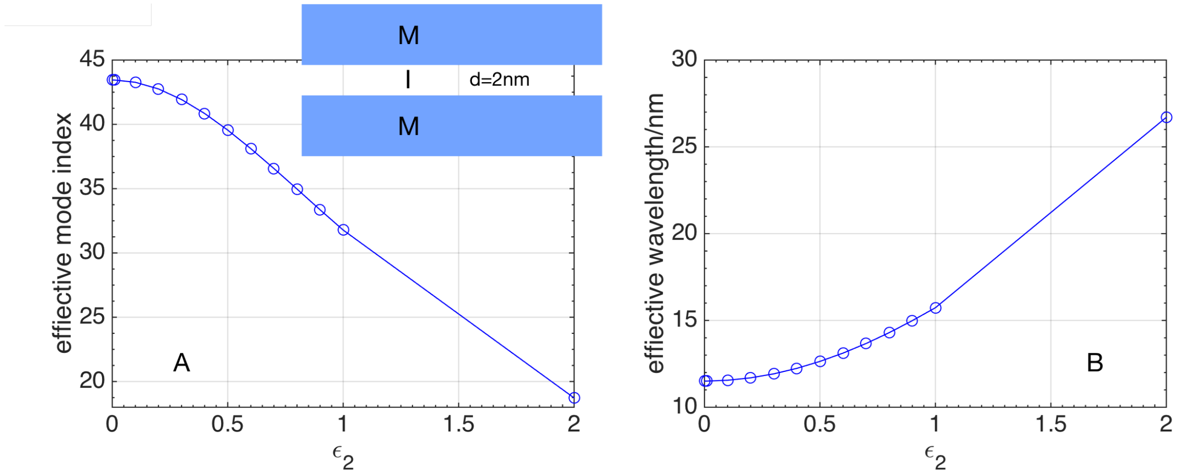

The effective mode index (A) and shortest wavelength (B) vs the loss ε_2. We model a 2D MIM structure with a gap of 2nm. The dielectric function of the metal is modeled as $\varepsilon=-2-i\varepsilon_2$.

Supplementary Fig. 6:

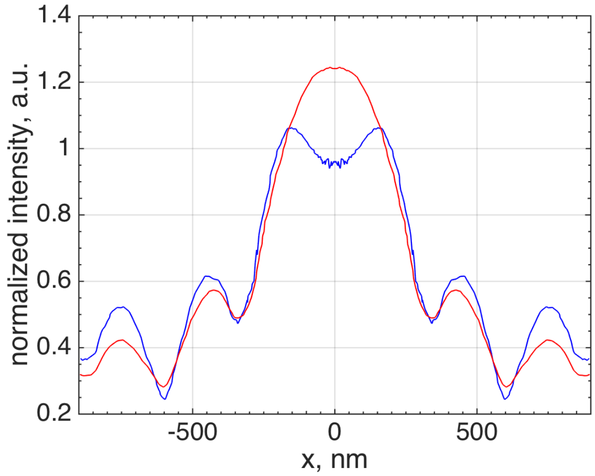

The effect of loss. High loss(red) vs low loss(blue).

Supplementary Movie (m1.wmv)
This video illustrates how surface waves interfere and form a super-resolution image of a point source. The structure consists of a lossless metal sphere (dielectric constant of ε=-2+0.01i, and permittivity μ=1) with diameter of 2μm placed 5nm above an infinite large lossless metal plate. A point dipole source is placed in the gap, 16nm away in X direction away from the center of the gap. The polarization of the dipole is in Z direction. A Finite-Time-Domain-Difference (FDTD) method is used for the simulation. The highest frequency used in the simulation corresponds to light with wavelength of 500nm in vacuum. The video shows a XZ cross-section plot of the intensity of all polarizations components of the E field. One can see that the surface waves propagate from the bottom of the sphere to the top. A white line marks the North Pole. The most constructive interference appears near the North Pole, forms an image of the point source. One may also notice that the adiabatic decompression process from the increasing wavelength of the surface waves. Even though the point source is only 16nm away from the South Pole of the sphere, which is well below the resolution limit. The displacement of this point source is clearly resolvable at the North Pole, illustrating the super-resolution power.

Supplementary Note:
Surface Wave on a Spherical Interface

We consider a spherical surface of radius R. A scalar wave would satisfy the following equation:

$$\nabla^2 E(r,\theta,\phi)+k_0^2\varepsilon(r)E(r,\theta,\phi)=0 \tag{0.1}$$

We consider a solution of the form:

$$E(r,\theta,\phi)=A(r,\theta)e^{im\phi} \tag{0.2}$$

Replacing (0.2) in (0.1) we obtain:

$$\frac{1}{r^2}\frac{\partial}{\partial r}\left(r^2\frac{\partial A}{\partial r}\right)+\frac{1}{r^2\sin\theta}\frac{\partial}{\partial\theta}\left(\sin\theta\frac{\partial A}{\partial\theta}\right)+\left[k_0^2\varepsilon(r)-\frac{m^2}{r^2\sin^2\theta}\right]A=0 \tag{0.3}$$

Let us assume a separable solution:

$$A(r,\theta)=F(r)U(R\theta) \tag{0.4}$$

Notice that in the argument of the function U we have included the radius of curvature, so that we have the length along the interface $s=R\theta$. Using (0.4) we obtain:

$$\frac{1}{F(r)}\frac{\partial}{\partial r}\left(r^2\frac{\partial F(r)}{\partial r}\right)+r^2k_0^2\varepsilon(r)=-\frac{1}{U(R\theta)\sin\theta}\frac{\partial}{\partial\theta}\left(\sin\theta\frac{\partial U(R\theta)}{\partial\theta}\right)+\frac{m^2}{\sin^2\theta} \tag{0.5}$$

It is convenient to express (0.5) in terms of $s=R\theta$:

$$\frac{1}{F(r)}\frac{d}{dr}\left(r^2\frac{dF(r)}{dr}\right)+r^2k_0^2\varepsilon(r)=-\frac{R^2}{U(s)\sin(s/R)}\frac{d}{ds}\left[\sin\left(\frac{s}{R}\right)\frac{dU(s)}{ds}\right]+\frac{m^2}{\sin^2(s/R)} \tag{0.6}$$

Expanding the right hand side:

$$\frac{1}{R^2F(r)}\frac{d}{dr}\left(r^2\frac{dF(r)}{dr}\right)+\frac{r^2k_0^2\varepsilon(r)}{R^2}=-\frac{1}{U(s)}\left[\frac{d^2U(s)}{ds^2}+\frac{\frac{1}{R}}{\tan\left(\frac{s}{R}\right)}\frac{dU(s)}{ds}\right]+\frac{\left(\frac{m}{R}\right)^2}{\sin^2\left(\frac{s}{R}\right)} \tag{0.7}$$

By introducing the surface wavevector k_s, equation (0.7) separates as follows:

$$\frac{1}{R^2}\frac{d}{dr}\left(r^2\frac{dF(r)}{dr}\right)+\left[\frac{r^2k_0^2\varepsilon(r)}{R^2}-k_s^2\right]F(r)=0 \tag{0.8}$$

$$\frac{d^2U(s)}{ds^2}+\frac{\frac{1}{R}}{\tan\left(\frac{s}{R}\right)}\frac{dU(s)}{ds}+\left[k_s^2-\frac{\left(\frac{m}{R}\right)^2}{\sin^2\left(\frac{s}{R}\right)}\right]U(s)=0 \tag{0.9}$$

In the limit of infinite radius of curvature $R\to\infty$, the second of (0.9) reduces to:

$$\frac{d^2U(s)}{ds^2}+\frac{1}{s}\frac{dU(s)}{ds}+\left[k_s^2-\frac{m^2}{s^2}\right]U(s)=0 \tag{0.10}$$

Equation (0.10) may be recognized as the Bessel equation and has solutions in terms of cylindrical waves:

$$U(s)=aJ_m(k_ss)+bY_m(k_ss) \qquad ; \quad s\geq 0 \tag{0.11}$$

for $R\to\infty$ we must recover the plasmon at a planar interface, so $k_s=k_0\sqrt{\frac{\varepsilon_d\varepsilon_m}{\varepsilon_d+\varepsilon_m}}=k_p$;

For the general case we have:

$$U(s) = a\,P^{m}_{\frac{\sqrt{1+4k_s^2R^2}-1}{2}}\left[\cos\left(\frac{s}{R}\right)\right] + b\,Q^{m}_{\frac{\sqrt{1+4k_s^2R^2}-1}{2}}\left[\cos\left(\frac{s}{R}\right)\right] \qquad ; \quad 0 \le s \le \pi R \qquad (0.12)$$

The degree of the associated Legendre function must be integer to ensure the continuity of the field at $s = \pi R$, so $k_s^2 R^2 = n(n+1)$. The coefficient b must also vanish to keep the solution finite everywhere:

$$U(s) = a\,P_n^m\left[\cos\left(\frac{s}{R}\right)\right] \qquad ; \quad 0 \le s \le \pi R \qquad (0.13)$$

For a large sphere the solution (0.13) should approach (0.11), and that occurs for:

$$n \approx 1 + \text{int}\left[k_p R\right] = 1 + \text{int}\left[k_0 R \sqrt{\frac{\varepsilon_d\, \varepsilon_m}{\varepsilon_d + \varepsilon_m}}\right] \qquad (0.14)$$

Double Sphere System

To prove the proposed super-resolution imaging mechanism in the case of two spheres, which are not in contact, we use a method-of-moment (MoM) software (FEKO 6.0) for numerical simulation. The system consists of two metal spheres with radius of 1.25 μm placed 5 nm apart (supplementary Fig3a). To demonstrate the proposed imaging mechanism, we use a perfect metal (dielectric constant of -2+0.001i) to model the system. The effect of loss will be discussed later. The surface plasmon polaritons, excited by a point dipole placed at the middle of the gap, exhibit adiabatic decompression. All of the rays launched from a point dipole at the South Pole travel by an equal optical path to exhibit constructive interference at the North Pole. If the point dipole is moved away from the South Pole, the corresponding image point generated from interference will move by an equal optical distance from the North Pole. However, the physical distance that corresponds to the displaced optical distance is much larger. As shown in supplementary Fig. 3b, an 8 nm displacement of a point source in the gap can be well resolved at the North Pole, which is 40 times beyond the diffraction limit of 250 nm in this system. The resolution depends on the size of the gap, increasing the gap distance reduces the resolution. Supplementary Fig. 3c shows that, if we increase the gap size to 16nm, the displacement of the image of the same point dipole becomes hard to resolve. To further help visualize this super-resolution imaging process; a supplementary animation is created by a finite-difference-time-domain (FDTD) simulation showing how the surface wave interferes to form a super-resolution image. The adiabatic decompression depends critically on the smoothness of the surface, which is critical to preserve the continuity of phase relation of light. Any roughness comparable to the local wavelength will induce strong light scattering and violate the adiabatic condition.

Green's function for the inhomogeneous index

We look for the Green's function in the double surface case:

$$\begin{cases} \dfrac{d^2U(s)}{ds^2}+\dfrac{1}{R\tan(s/R)}\dfrac{dU(s)}{ds}+\left[k_p^2-\left(\dfrac{m}{R}\right)^2\right]\dfrac{U(s)}{\sin^2(s/R)}=\delta\left[\cos\left(\dfrac{s}{R}\right)-\cos\left(\dfrac{s_0}{R}\right)\right] & ;\ 0<s\le\dfrac{\pi R}{2} \\ \dfrac{d^2U(s)}{ds^2}+\dfrac{1}{R\tan(s/R)}\dfrac{dU(s)}{ds}+\left[k_p^2-\dfrac{m^2}{R^2\sin^2(s/R)}\right]U(s)=0 & ;\ \dfrac{\pi R}{2}<s\le\pi R \end{cases} \tag{2.1}$$

The problem (2.1) can be cast in the Sturm-Liouville canonical form using the following procedure. We write (2.1) as:

$$A(s)\frac{d^2U(s)}{ds^2}+B(s)\frac{dU(s)}{ds}+C(s)U(s)=S(s)$$

$$\begin{cases} S(s)=\delta\left[\cos\left(\dfrac{s}{R}\right)-\cos\left(\dfrac{s_0}{R}\right)\right] \\ A(s)=1 \\ B(s)=\dfrac{1}{R\tan(s/R)} \\ C(s)=\left[\dfrac{k_p^2R^2-m^2}{R^2\sin^2(s/R)}\right]u_0\left(\dfrac{\pi}{2}R-s\right)+\left[k_p^2-\dfrac{m^2}{R^2\sin^2(s/R)}\right]u_0\left(s-\dfrac{\pi}{2}R\right) \end{cases} \tag{2.2}$$

From these we can recover the canonical Sturm-Liouville form:

$$\frac{d}{ds}\left\{p(s)\frac{d}{ds}U(s)\right\}-q(s)U(s)=f(s)$$

$$\begin{cases} p(s)=\exp\left[\displaystyle\int_0^s\frac{B(x)}{A(x)}dx\right]=\exp\left[\displaystyle\int_0^s\frac{1}{R\tan(x/R)}dx\right]=\sin\left(\dfrac{s}{R}\right) \\ q(s)=-p(s)\dfrac{C(s)}{A(s)}=-\left[\dfrac{k_p^2R^2-m^2}{R^2\sin(s/R)}\right]u_0\left(\dfrac{\pi}{2}R-s\right)+\left[\dfrac{k_p^2R^2\sin^2\left(\dfrac{s}{R}\right)-m^2}{R^2\sin(s/R)}\right]u_0\left(s-\dfrac{\pi}{2}R\right) \\ f(s)=p(s)\dfrac{S(s)}{A(s)}=\sin\left(\dfrac{s}{R}\right)\delta\left[\cos\left(\dfrac{s}{R}\right)-\cos\left(\dfrac{s_0}{R}\right)\right] \end{cases} \tag{2.3}$$

In order to find an orthonormal set of basis functions for the Sturm-Liouville problem it is convenient to treat the wavevector k_p as an eigenvalue parameter and we replace it by $n(n+1)$. Therefore we consider the homogeneous version of (2.3) and we redefine $q(s)$ and a weight function $\sigma(s)$:

$$\frac{d}{ds}\left\{p(s)\frac{d}{ds}U(s)\right\}-q(s)U(s)-n(n+1)\sigma(s)U(s)=0$$

$$\begin{cases} q(s)=\dfrac{m^2}{R^2\sin(s/R)} \\ \sigma(s)=-\sin\left(\dfrac{s}{R}\right)-\left[\dfrac{\cos\left(\dfrac{s}{R}\right)}{\tan(s/R)}\right]u_0\left(\dfrac{\pi}{2}R-s\right)= \\ =-\sin\left(\dfrac{s}{R}\right)u_0\left(s-\dfrac{\pi}{2}R\right)-\dfrac{1}{\sin\left(\dfrac{s}{R}\right)}u_0\left(\dfrac{\pi}{2}R-s\right) \end{cases} \tag{2.4}$$

From the Sturm-Liouville theory we know that the solutions of the eigenvalue problem (2.4) form an orthogonal basis with respect to the weight function $\sigma(s)$ defined in (2.4). The solutions of the eigenvalue problem (2.4) are the following:

$$\phi_{nm}(s)=L_{nm}P_n^m\left[\cos\left(\frac{s}{R}\right)\right]u_0\left(s-\frac{\pi R}{2}\right)+$$

$$+\left\{a\cos\left[\sqrt{n(n+1)-m^2}\log\left(\tan\frac{s}{2R}\right)\right]+b\sin\left[\sqrt{n(n+1)-m^2}\log\left(\tan\frac{s}{2R}\right)\right]\right\}u_0\left(\frac{\pi R}{2}-s\right)$$

$$\begin{cases} L_{nm}=\sqrt{\dfrac{2n+1}{4\pi}}\sqrt{\dfrac{(n-m)!}{(n+m)!}} \\ a=P_n^m(0) \\ b=\left(\dfrac{2m-n}{2\sqrt{n(n+1)-m^2}}\right)P_{(n+1)}^m(0) \end{cases}$$

(2.5)

The functions (2.5) are orthogonal with respect to the weight function $\sigma(s)$:

$$\int_0^{\pi R}\phi_{n_1m}(s)\phi_{n_2m}(s)\sigma(s)ds=\delta_{n_1n_2} \tag{2.6}$$

Note: The Green's function can also be obtained in closed form from the knowledge of a solution $U_1(s)$ for $s<s_0$ and another solution $U_2(s)$ for $s>s_0$ as follows:

$$G_m(s,s_0)=\begin{cases} \dfrac{U_2(s_0)}{p(s_0)W(s_0)}U_1(s) \ ; \quad a<s<s_0 \\ \dfrac{U_1(s_0)}{p(s_0)W(s_0)}U_2(s) \ ; \quad s_0<s<\pi \end{cases} \tag{2.7}$$

Nonlocal Response

We test if the proposed adiabatic decompression exists when the nonlocal effects on strongly confined plasmonic modes are taken into consideration. Once the gap between the two sphere reduces to a level comparable to the inter electronic distance, typically on the order of 0.1 nm, the quantum effect of strong electron− electron interactions in the dielectric response of metals becomes pronounced[1], which manifest itself as increased effective wavelength of the EM field in the gap region and a spatially dependent dispersion relation[1-17]. Our simulations so far have been relied on a localized picture of the dielectric response of metallic materials, since the gap size is larger than 2nm.

Recently, Luo *et al*[8] have developed an elegant method to incorporate the nonlocal response with standard computer simulation methods. They showed that the nonlocal response could be modeled by as a composite material, comprising a thin, ~0.1nm, dielectric layer on top of a local metal. This method allows us to examine the impact of nonlocal response with a FEM simulation on a 2D double sphere system. Supplementary Fig. 4 presents the result. As we have expected, in the case of a gap size of 2nm, the decompression remains, even when the nonlocal effect is included.

Loss

The resolution of an adiabatic lens depends on the shortest wavelength of light that the MIM (metal-insulator-metal) structure can compress to, which is critically determined by the level of confinement of light inside the gap region. This could be understood from the hyperbolic dispersion relationship of surface plasmon inside a MIM waveguide, $k_{x,y}^2 - k_z^2 = k_0^2$, where k_z is proximally the skin depth. Therefore the highest k_{xy} is about the skin depth.

To further investigate the effect of loss, we performed a simulation of a 2D MIM structure (Supplementary Fig. 5). We model a 2D MIM structure with a gap of 2 nm. The dielectric function of the metal is modeled as $\varepsilon = -2 - i\varepsilon_2$. The figure shows that as the loss increase, (ε_2 increase), the effective mode index decrease and the shortest compressible wavelength increase sas well. Since the resolution of an adiabatic lens is determined by the ratio between the wavelength of the EM wave at the far field and that of near field, the decrease of the effective mode index reduces the maximum magnification as well as the resolution.

The loss directly impacts the resolution of the proposed adiabatic lens. Supplementary Fig. 6 shows a profile of two non-coherent dipoles. The red curve corresponds to the high loss case of dielectric constants of -2+i, while the blue one corresponds to the low loss case. The images of two dipoles are resolvable (blue) when the loss is low, and not resolvable when high (red).

Supplementary Reference:

1 Scholl, J. A., Koh, A. L. & Dionne, J. A. Quantum plasmon resonances of individual metallic nanoparticles. *Nature* **483**, 421-427 (2012).

2 Apell, P. & Ljungbert, Å. A general non-local theory for the electromagnetic response of a small metal particle. *Physica Scripta* **26**, 113 (1982).

3 Apell, P., Ljungbert, Å. & Lundqvist, S. Non-local optical effects at metal surfaces. *Physica Scripta* **30**, 367 (1984).

4 Chang, R. & Leung, P. Nonlocal effects on optical and molecular interactions with metallic nanoshells. *Physical Review B* **73**, 125438 (2006).

5 Ciracì, C. *et al.* Probing the ultimate limits of plasmonic enhancement. *Science* **337**, 1072-1074 (2012).

6 Fernández-Domínguez, A., Wiener, A., García-Vidal, F., Maier, S. & Pendry, J. Transformation-optics description of nonlocal effects in plasmonic nanostructures. *Physical Review Letters* **108**, 106802 (2012).

7 García de Abajo, F. J. Nonlocal effects in the plasmons of strongly interacting nanoparticles, dimers, and waveguides. *The Journal of Physical Chemistry C* **112**, 17983-17987 (2008).

8 Luo, Y., Fernandez-Dominguez, A., Wiener, A., Maier, S. A. & Pendry, J. Surface plasmons and nonlocality: A simple model. *Physical Review Letters* **111**, 093901 (2013).

9 McMahon, J. M., Gray, S. K. & Schatz, G. C. Nonlocal optical response of metal nanostructures with arbitrary shape. *Physical Review Letters* **103**, 097403 (2009).

10 McMahon, J. M., Gray, S. K. & Schatz, G. C. Optical properties of nanowire dimers with a spatially nonlocal dielectric function. *Nano letters* **10**, 3473-3481 (2010).

11 Mortensen, N. A., Raza, S., Wubs, M., Søndergaard, T. & Bozhevolnyi, S. I. A generalized non-local optical response theory for plasmonic nanostructures. *Nature Communications* **5** (2014).

12 Raza, S., Toscano, G., Jauho, A.-P., Wubs, M. & Mortensen, N. A. Unusual resonances in nanoplasmonic structures due to nonlocal response. *Physical Review B* **84**, 121412 (2011).

13 Romero, I., Aizpurua, J., Bryant, G. W. & García De Abajo, F. J. Plasmons in nearly touching metallic nanoparticles: singular response in the limit of touching dimers. *Optics Express* **14**, 9988-9999 (2006).

14 Teperik, T. V., Nordlander, P., Aizpurua, J. & Borisov, A. G. Robust subnanometric plasmon ruler by rescaling of the nonlocal optical response. *Physical Review Letters* **110**, 263901 (2013).

15 Toscano, G., Raza, S., Jauho, A.-P., Mortensen, N. A. & Wubs, M. Modified field enhancement and extinction by plasmonic nanowire dimers due to nonlocal response. *Optics Express* **20**, 4176-4188 (2012).

16 Toscano, G. *et al.* Nonlocal response in plasmonic waveguiding with extreme light confinement. *Nanophotonics* **2**, 161-166 (2013).

17 Wiener, A., Fernández-Domínguez, A. I., Horsfield, A. P., Pendry, J. B. & Maier, S. A. Nonlocal effects in the nanofocusing performance of plasmonic tips. *Nano Letters* **12**, 3308-3314 (2012).
